# Using the PubAnnotation ecosystem to perform agile text mining on *Genomics & Informatics*: a tutorial review

**DOI:** 10.5808/GI.2020.18.2.e13

**Published:** 2020-06-16

**Authors:** Hee-Jo Nam, Ryota Yamada, Hyun-Seok Park

**Affiliations:** 1 Bioinformatics Laboratory, ELTEC College of Engineering, Ewha Womans University, Seoul 03760, Korea; 2 Fuku Corporation, Tokyo 113-0033, Japan; 3 Center for Convergence Research of Advanced Technologies, Ewha Womans University, Seoul 03760, Korea

**Keywords:** named entity recognition, natural language processing, text mining

## Abstract

The prototype version of the full-text corpus of *Genomics & Informatics* has recently been archived in a GitHub repository. The full-text publications of volumes 10 through 17 are also directly downloadable from PubMed Central (PMC) as XML files. During the Biomedical Linked Annotation Hackathon 6 (BLAH6), we experimented with converting, annotating, and updating 301 PMC full-text articles of *Genomics & Informatics* using PubAnnotation, a system that provides a convenient way to add PMC publications based on PMCID. Thus, this review aims to provide a tutorial overview of practicing the iterative task of named entity recognition with the PubAnnotation/PubDictionaries/TextAE ecosystem. We also describe developing a conversion tool between the Genia tagger output and the JSON format of PubAnnotation during the hackathon.

## Introduction

*Genomics & Informatics* is the official journal of the Korea Genome Organization. The prototype version of the full-text corpus of *Genomics & Informatics* (GNI version 1.0) has recently been archived in a GitHub repository [1,2]. Further preprocessing and semi-automatic editing are underway to prepare the next version of GNI. As the volume numbers of *Genomics & Informatics* are growing, we needed a persistent and sharable repository to annotate and to upload the PMC articles of *Genomics & Informatics*.

During the Biomedical Linked Annotation Hackathon 6 (BLAH6), we experimented with annotating the PMC articles of *Genomics & Informatics*, making a custom dictionary using PubDictionaries, and uploading the annotation results into PubAnnotation. PubDictionaries is a public repository of dictionaries and PubAnnotation is a public repository of text annotations; these resources are primarily developed and maintained by the Database Center for Life Science (DBCLS), Japan [3,4]. PubAnnotation and PubDictionaries adopt a dictionary-based agile text mining approach, wherein iterative development cycles can be carried out by modifying a dictionary, manually reannotating, and automatically reannotating [5].

Thus, the purpose of this interdisciplinary tutorial review is to share our experiences of using the PubAnnotation ecosystem and writing a conversion script to apply to the *Genomics & Informatics* corpus [6,7]. We provide an introductory overview to briefly introduce basic information extraction tasks and dictionary-based named entity recognition (NER) for non-experts in the field, and to provide some helpful pointers to start a deeper investigation into agile text mining and corpus annotation techniques in general.

The conversion code between the Genia tagger output and JSON files and the indexed XML files for Genomics & Informatics during BLAH6 are both available through GitHub (https://github.com/Ewha-Bio/Genomics-Informatics-Corpus/tree/master/code/BLAH; https://github.com/Ewha-Bio/Genomics-Informatics-Corpus/tree/master/XML).

## Creating a PubAnnotation Pilot Project

PubAnnotation supports an agile approach to text mining by instantiating software components that allow for decomposed parallel development, while also facilitating continuous integration [5].

The PubAnnotation ecosystem is designed to be an open, API-driven system, and to harness changes for the user’s advantage. Annotations can be obtained from an external web service, which is called an annotation server. Through this principle, potential users are able to use the system to fine-tune and adjust their existing project [8].

During BLAH6, we created a PubAnnotation pilot project, called BLAH6-GNI-Corpus (http://pubannotation.org/projects/BLAH6-GNI-Corpus), initially to upload the *Genomics & Informatics* corpus. PubAnnotation provides a convenient way to add, annotate, and edit PMC publications based on PMCID. We specified the PMCID and uploaded the text files of *Genomics & Informatics*. In total, 301 documents were imported into the project.

**Fig. 1.**
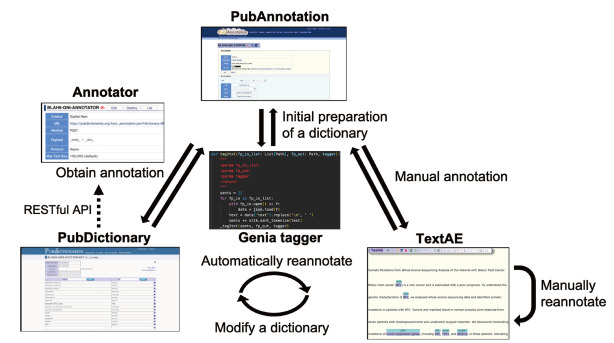
An Agile approach to text mining with PubAnnotation, PubDictionaries, and TextAE.

Three components were used to implement the iterations of agile development, as shown in Fig. 1: PubAnnotation, a storage component for regression testing; TextAE, a manual annotation tool; and PubDictionaries, a dictionary-based annotator. These three components of the PubAnnotation ecosystem provide many ways to proceed with NER projects. The following sections provide one scenario, in which we conducted agile text mining with these components by adding the PMC publications of *Genomics & Informatics* to a PubAnnotation project, writing a script to upload the existing tagged documents, creating a PubDictionaries project to obtain annotations, and editing the annotations manually with TextAE [5].

## A Tutorial Example

We initially used the GENIA tagger to annotate biological terms when developing the GNI corpus 1.0 [9,10]. It is easiest to understand how PubAnnotation and PubDictionaries might be used to integrate the GNI corpus 1.0 on the basis of an example.

### An exemplary output format of the GENIA tagger

**Fig. 2.**
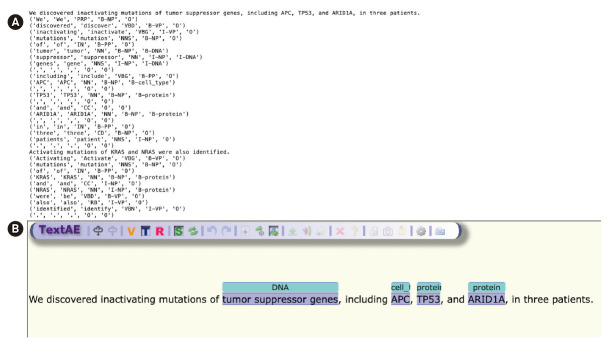
(A) Initial NER result by GENIA tagger. (B) A visualization of NER generated by TextAE.

The annotation result of PMCID 6440663—“We discovered inactivating mutations of tumor suppressor genes, including APC, TP53, and ARID1A, in three patients.”—as shown in Fig. 2, is used as an example sentence.

The GENIA tagger outputs the base forms, part-of-speech (POS) tags, chunk tags, and named entity tags. The tagger is specifically tuned for biomedical texts such as MEDLINE abstracts. Fig. 2A is a direct output from the GENIA tagger, and 2B is a visualization of NER generated by TextAE [10], the default viewer and editor of PubAnnotation. Four different levels of tags are attached for each word in the example sentence: base forms, POS tags, chunk tags, and named-entity tags. For example, “TP53”, “TP53”, “NN”, “B-NP”, and “B-protein” indicate that the part of speech of the word “TP53” is a noun (‘NN’), that the word begins a noun phrase (‘B-NP’), and that it begins a phrase of a protein name (“B-protein”).

The last tag is a semantic-level tag to classify named entities in the text into pre-defined categories such as proteins, DNAs, RNAs, cell lines, and cell types. For named-entity tags, B/I/O notation was used, wherein the B/I/O terminology refers to the beginning of the phrase (B), internal to the phrase (I), and outside of the phrase (O).

Fig. 2 shows that APC was wrongly classified, because “APC” could refer to the adenomatous polyposis coli gene or to an antigen-presenting cell. Generally, biomedical NER faces difficulties for many reasons, prominent among which are the often-ambiguous abbreviations that are frequently used in the biomedical field.

### Wring a Python script to convert GENIA tagging results into a PubAnnotation format

**Fig. 3.**
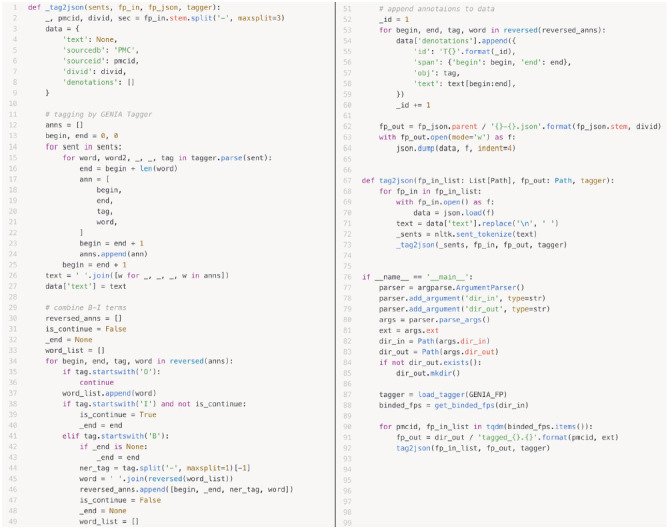
A conversion tool written in Python.

The desired result of a dictionary-based text annotation task would be an index of the dictionary entities corresponding to the referenced target texts. PubAnnotation’s text sequencer turns a document into a sequence of characters, so that positions in the document can be specified unambiguously by character offsets. For this reason, a conversion tool between the Genia tagger output and the JSON import/export format of PubAnnotation was written in Python during BLAH6. As shown in Fig. 3, we extracted the text field from a JSON file, and tokenized it by sentence, using the Natural Language Toolkit (NLTK) package, as in lines 67–73 [11]. This tokenizer divides a text into a list of sentences by using an unsupervised algorithm to build a model for abbreviation words, collocations, and words that start sentences. In line 12–27, indexes for each word in the Genia output are calculated by adding up the white spaces and character lengths. In lines 29–60, a new list is created, containing the begin index, end index, named entity tag, and word; B-tags and I-tags are combined, and the indexes are recalculated. Finally, the list to the denotation field of the dictionary is appended and converted into JSON.

### Manual editing using TextAE

A facility of visualization and manual editing is one of the primary aspects of making the PubAnnotation ecosystem adoptable by end-users. A user can easily add a new entry or delete an entry, in a try-and-revise manner.

**Fig. 4.**
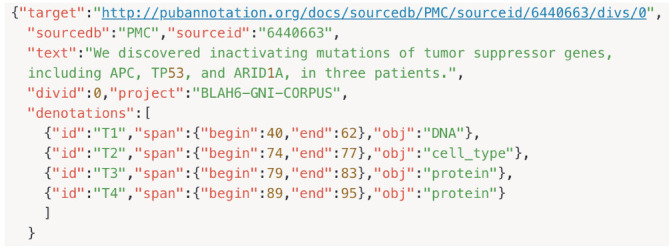
PubAnnotation JSON format with its tag and span indexing information.

In Fig. 4, there are four denotations for our example sentence, T1 through T4, with its tag and span information. The first one connects span 40-62 (the text spanning from the 40th to 62nd characters) to DNA, while the fourth connects span 89-95 to Protein. The default interpretation of T4 is as follows: the text span between *"span":{"begin":89, "end":95}* denotes an entity *T1 "id":"T1"* of which the type is *Protein*.

**Fig. 5.**
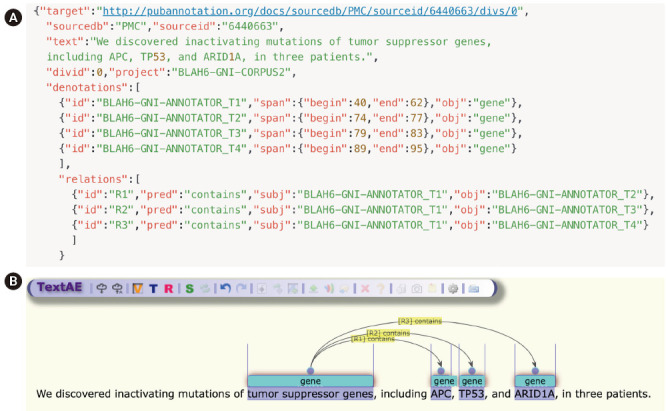
(A) JSON format of the example sentence. (B) A visualization of named entity recognition generated by TextAE.

Once an annotation file is prepared, TextAE can be used for manual editing of NER [12], as in Fig. 5. TextAE is a web-based graphical annotation editor, which was developed as an open-source project. APC is now tagged as a “gene,” as shown in Fig. 5, after manual editing. In addition to NER tagging, the example also presents the ease of using TextAE for manual editing of relation annotations, showing that the two entities, T1 and T2, that are introduced by the two denotations, are related to each other by the predicate “contains”, specified by the two different keys, so the relationship is directional.

## Summary

In this tutorial review, we presented our experiences of conducting and agile text mining. During BLAH6, we created two separate PubAnnotation projects (BLAH6-GNI-Corpus and BLAH6-GNI-Corpus2), a dictionary (BLAH6-GNI-Dictionary), and an annotator (BLAH6-GNI-Annotator). A total of 12,908 labels were registered in the PubAnnotation ecosystem (http://pubannotation.org/annotators/BLAH6-GNI-ANNOTATOR).

While developing a conversion tool during BLAH6, indexing and calculating spans was a non-trivial task, as PubAnnotation utilizes character-based indexing; enforcement of a fixed tokenization of the text is technically expensive.

Some minor suggestions relate to the user interface. In some menus, it was not fully obvious for a first-time user of the system what was clickable. We also had to create two separate projects, simply to utilize PubAnnotation’s text sequencer.

We assume that there are many categories of users with different levels of experience and familiarity with PubAnnotation, ranging from pure natural language processing specialists to biomedical research end users. We hope that some additional features will be added to the PubAnnotation ecosystem, to provide diversified access to different groups of users, who have different needs regarding workflow and information density.
